# *Scedosporium apiospermium* keratitis: a case report

**DOI:** 10.1186/s13256-022-03315-9

**Published:** 2022-03-04

**Authors:** Umut Karaca

**Affiliations:** Present Address: Faculty of Medicine Department of Ophthalmology, Suleyman Demirel University, Isparta, Turkey

**Keywords:** *Scedosporium* infection, Mycotic keratitis, Penetrating keratoplasty, Contact lens associated

## Abstract

**Background:**

*Scedosporium apiospermum*, an opportunistic and filamentous fungus, is a rarely seen ocular entity that is difficult to identify and heal. We report a challenging case of *S. apiospermium* keratitis and discuss the treatment modalities in light of previous studies.

**Case presentation:**

A 30-year-old Turkish farmer with a history of contact lens misuse presented to our clinic with a painful corneal abscess and severe vision loss in his left eye. *S. apiospermum* was identified by spectrophotometric analysis. The patient was successfully treated with therapeutic penetrating keratoplasty, but was resistant to fluconazole and amphotericin B and susceptible but unresponsive to voriconazole.

**Conclusion:**

*S. apiospermum* keratitis should be considered in the differential diagnosis of immunocompromised and immunocompetent patients with history of ocular trauma and contact lens use, especially those who do not respond to treatment.

## Introduction

Fungal keratitis (also known as mycotic keratitis or keratomycosis), which is usually characterized by corneal epitheliopathy and stromal infiltration, especially with *Fusarium* and *Aspergillus* species, remains a challenge for ophthalmologists worldwide. It is more common in agricultural and developing countries, with an estimated global prevalence of approximately 1–1.2 million cases annually [1]. Nepal is a worsening example, with an estimated prevalence of 73/100,000, 27–62% of which is microbial keratitis, while in Turkey, the estimated incidence of fungal keratitis is 33/100,000 annually [2, 3]. Ocular trauma is the most common predisposing risk factor, particularly during vegetative contamination.

Pathogenic *Scedosporium* species, including *Scedosporium prolificans* and *Scedosporium apiospermum*, cause a wide range of clinical manifestations, from subclinical infection to severe respiratory disease, especially colonization of the respiratory tract [4]. *S. apiospermum* (also known as the asexual form of the ascomycete *Pseudallescheria boydii*) is a significant opportunistic pathogen that causes serious infections with very high levels of antifungal resistance [5]. Keratomycosis, scleritis, and endophthalmitis are the most common ocular involvements in immunocompromised patients [6]. In fact, *S. apiospermum* keratitis (the most common infection of *S. apiospermum* in immunocompetent patients) is uncommon, aggressive, and resistant to conventional antifungal therapies [5]. Here, we report a challenging case of *S. apiospermum* keratitis that was successfully treated with therapeutic penetrating keratoplasty. The keratitis was resistant to fluconazole and amphotericin B and susceptible to voriconazole. In addition, we discuss treatment modalities in light of previous studies.

## Case presentation

**Fig. 1:**
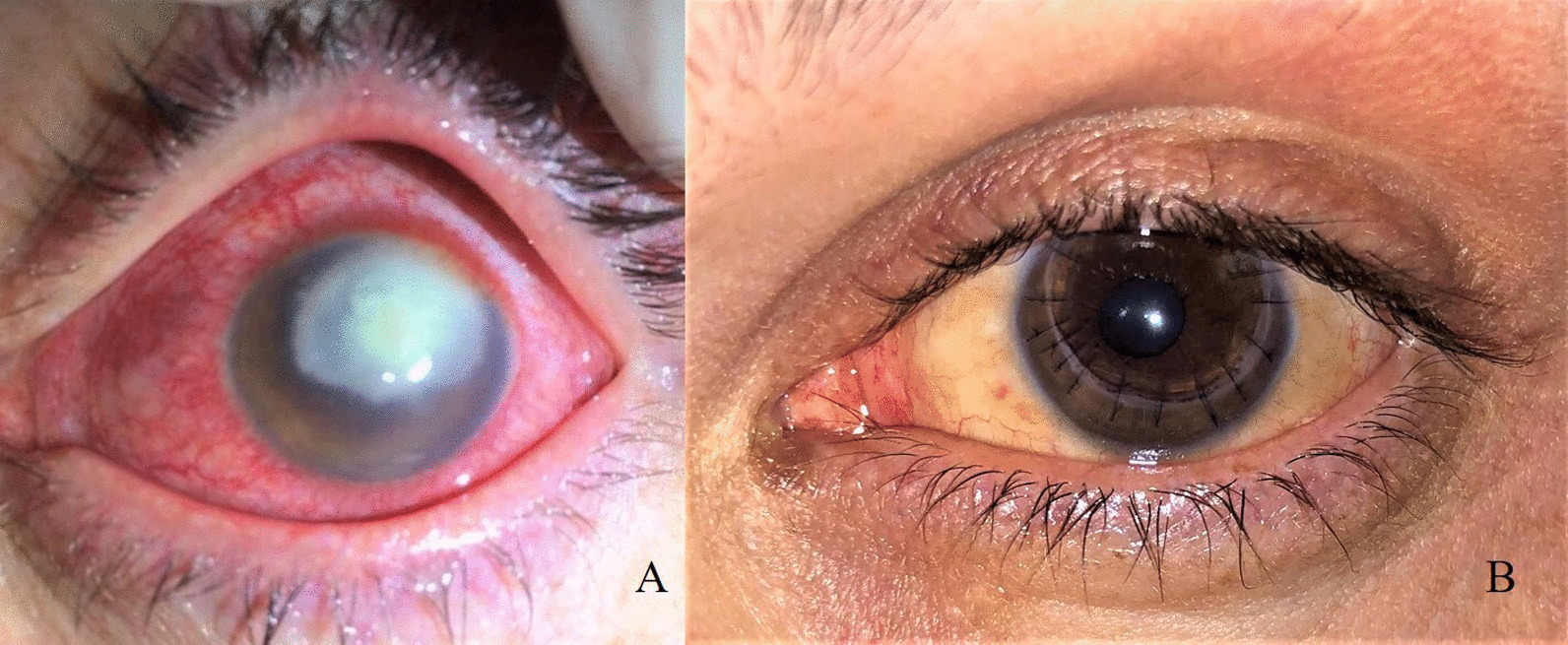
**A** Keratitis with excessive corneal abscess. **B** Postoperative first month of the patient.

A 30-year-old immunocompetent Turkish man presented to our clinic with a painful corneal abscess and severe vision loss in his left eye. His family and medical history showed no remarkable findings. The systemic examination results were not significant. He was a farmer and careless contact lens wearer. His eye symptoms started a month ago and had been treated with topical antibiotics and antiviral therapy for 2 weeks without microbiological examination. Due to disease progression despite treatment, he was referred to our tertiary ophthalmology clinic for further investigation and treatment. Visual acuity of the right eye was only light perception. Marked ciliary congestion and extensive corneal abscesses with corneal melting were observed. The anterior chamber structures were not visible because of excessive corneal abscesses (Fig. 1A). Corneal scrapings were taken immediately, and topical vancomycin (50 mg/ml), topical ceftazidime (50 mg/ml) hourly, and topical fluconazole (Fluzamed 0.3%, World Medicine, London, England) eight times daily were started immediately.

**Fig. 2:**
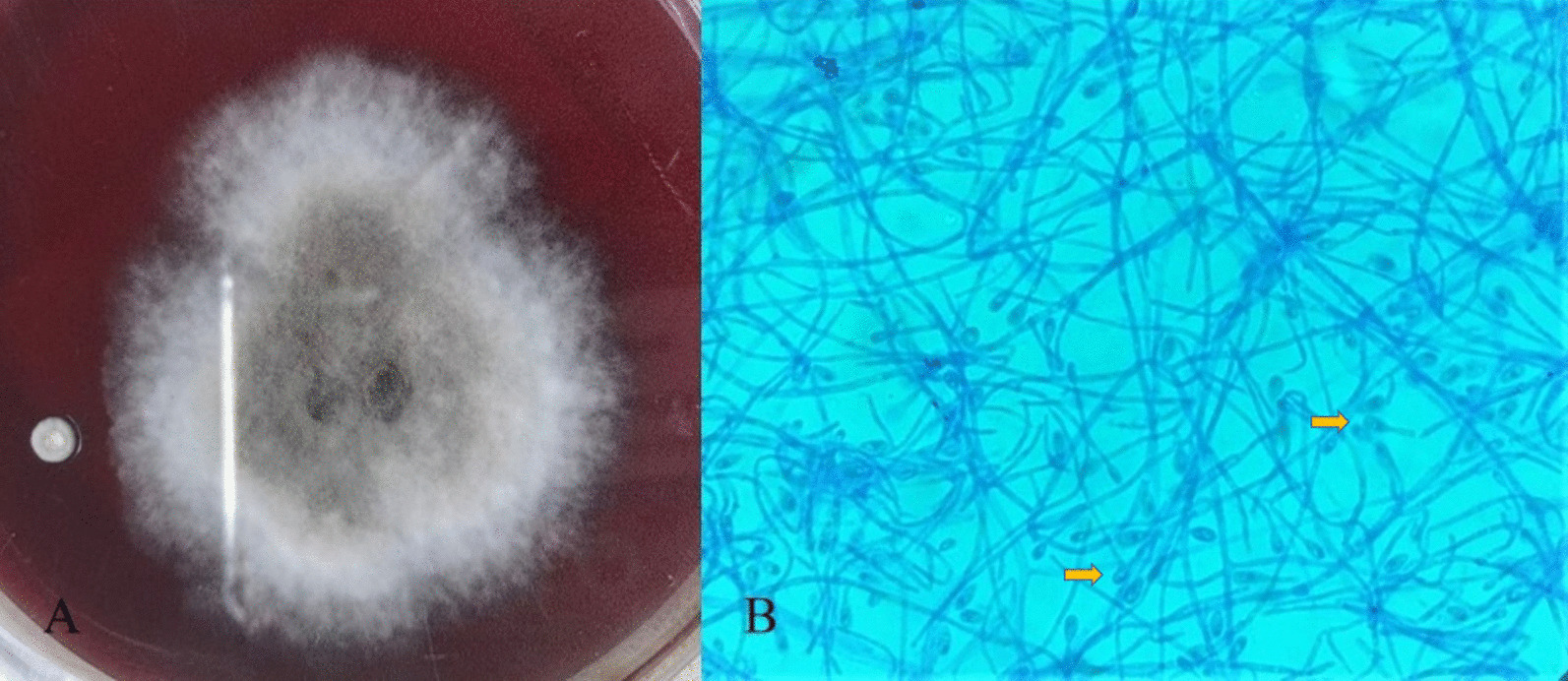
**A** Colony morphology of *Scedosporium apiospermum* on brain hard infusion agar. Colony appearances of the fungus were white to grayish on the margin with an olive-green umbonate center. **B** Microscopic appearance of *S. apiospermium* with lactophenol cotton blue stain. Septate hyphae with short or long slender conidiophores, bearing single conidia. The conidia were oval and unicellular, with the larger end toward the apex (arrows)

The microbiological samples for diagnostic procedures taken from corneal tissue, contact lens, and contact lens solution were inoculated onto Sabouraud dextrose agar (SDA) and brain hard infusion (BHI) agar on the day of admission. The plates were incubated at 37 °C. After 4 days of incubation, a phaeoid mold was observed on the plate, which was inoculated with a corneal tissue sample. No growth was observed in the other culture samples. The colony appearance of the fungus was white to grayish on the margin, with an olive green umbonate center and a woolly surface (Fig. 2A). The slide culture technique was used to examine the microscopic features of the fungal colonies. Direct microscopic preparation was performed using lactophenol cotton blue (LPCB) staining. The LPCB preparation showed septate hyphae with short or long slender conidiophores bearing single conidia. The conidia were oval and unicellular, with a larger end towards the apex (Fig. 2B). The mold was identified as *Scedosporium apiospermum* by matrix-assisted laser desorption ionization-time of flight mass spectrometry (MALDI TOF-MS) (Bruker Daltonics, Bremen, Germany). Antifungal susceptibility was studied using the gradient strip test and broth microdilution method at public health institutions. The isolate was resistant to fluconazole, amphotericin B, and miconazole, and sensitive to voriconazole.

Due to treatment unresponsiveness, fluconazole was switched to voriconazole on the third day of follow-up. Systemic voriconazole (Vfend 200 mg intravenously, Pfizer, USA) was added every 12 hours after the antifungal susceptibility test, as it was still unresponsive on the seventh day of follow-up. Despite adequate treatment, no clinical improvement was observed, and corneal melting was observed during follow-up. Therefore, surgical intervention was performed to remove infected tissue. Therapeutic penetrating keratoplasty was performed. The purulent material colonizing the anterior chamber was aspirated and sent for microbiological examination together with the corneal button preoperatively.

**Fig. 3:**
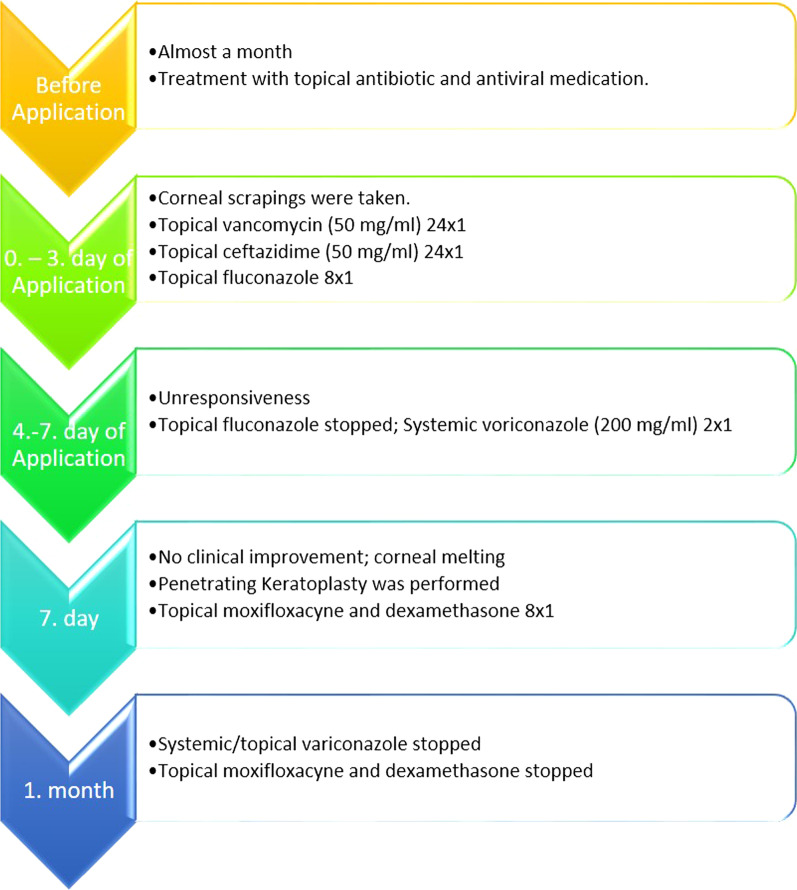
Treatment SAlgorithm of the patient

A week after the surgery, a well-formed anterior chamber with moderate corneal graft edema was observed. The visual acuity of the eye was 20/125. Oral and topical voriconazole with moxifloxacin and dexamethasone were maintained for a month after surgery (Fig. 1B). The treatment is summarized in Fig. 3.

Keratoplasty sutures were excised in the sixth month after surgery. The final visual acuity of the affected eye was 20/100, and a clear donor cornea and silent anterior chamber were examined during the second year of follow-up.

## Discussion

*S. apiospermum*, an opportunistic and filamentous fungus, is a rarely seen ocular entity that is difficult to identify and heal. In this case, we would like to highlight these remarkable features. First, long-term contact lens use or misuse may predispose an immunocompetent young patient to this disease. Additionally, identification is difficult because it mimics *Fusarium* and *Aspergillus* species. Finally, penetrating keratoplasty performed at the right time is important for disease healing and vision recovery.

Environmental conditions seem to have a strong influence on the occurrence of filamentous fungal keratitis (such as *Fusarium*, *Aspergillus*, and *Scedosporium*) [7]. Healthy young men who are usually engaged in agriculture or other outdoor work are the main risk group for filamentous keratitis. Trauma is a predisposing factor in most diseases because of the protective features of the intact epithelium. The use of contact lenses is prominent in the formation of chronic epitheliopathies, especially in *Fusarium* species [8]. In recent years, *Scedosporium* spp. have been encountered with increasing frequency. Although the risk factors and clinical manifestations are similar to filamentous keratitis, *Scedosporium* species can be distinguished from others by their typical microscopic appearance and resistance to topical antifungal treatment.

There are a large number of reports of serious *S. apiospermum* infections in immunocompromised patients worldwide. However, keratitis is the most common manifestation of *S. apiospermum* ocular infection in immunocompetent people and, in most cases, it is usually preceded by corneal injury or contact lens misuse [9, 10]. The number of contact lens wearers is estimated to be 140 million worldwide including 750,000 in our country, and is predicted to increase gradually. Therefore, *Scedosporium* spp. infections are thought to be encountered more frequently [11]. Lipofuscin-like microdeposits have been shown in corneal examinations of those who wear long-term contact lenses, as a result of chronic oxygen deprivation and microtrauma, which may be associated with keratitis susceptibility [12]. The long-term use of contact lenses by an agricultural laborer (proper cornea in the proper environment), as in our patient, paves the way for this type of infection.

The diagnosis of keratomycosis begins with the suspicion of a clinical appearance and is usually defined by morphological features in culture [7]. However, hyalinized hyphal septates on direct microscopic examination complicate the distinction of *Scedosporium* species from *Fusarium*, *Aspergillus*, and other relatively common hyaline hyphomycetes [4]. Therefore, identification tests that support traditional methods are utilized. Various molecular techniques, such as mass spectrometry, polymerase chain reaction, and DNA sequencing, are being used for exact fungal identification. In our case, MALDI-TOF-MS, a fast and reliable method for species identification of filamentous fungi, was used to identify *S. apiospermum* [13].

Despite scientific developments, it is difficult to control keratomycosis cases with antifungal therapy because of late diagnosis and antifungal resistance. It is not yet clear which medical treatment is optimal, how long it should be used for, and when it should be stopped. Although *S. apiospermum* is thought to have varying susceptibilities to voriconazole, posaconazole, and miconazole, combined therapies are particularly prominent, especially for late-diagnosed invasive keratomycosis. *In vitro* studies have shown that a combination of azole and echinocandin is the most effective against *Scedosporium* isolates. However, in the treatment of keratomycosis, combined therapy is challenging because of the difficulty in finding the eye drop form of an appropriate drug and the limited effect of systemic antifungal therapy on corneal tissue. We preferred to use both systemic and topical forms of voriconazole because of its broad antifungal spectrum. However, we were unable to reach the eye drop form of natamycin, caspofungin, or amphotericin B to combine.

Besides medical antifungal therapy, a literature search shows that patients undergoing penetrating keratoplasty are not rare [10, 14, 15]. The most common indications were melting and perforation. Surprisingly, in our case, voriconazole, which is a sensitive *in vitro* tests, could not prevent progression to necrotizing keratitis and melting, despite local and systemic administration. This can be explained by the spread of keratitis to all corneal layers, resulting in necrosis. Therefore, keratoplasty should be considered a treatment option in keratitis cases that have invaded all corneal layers, where it is not possible to regenerate the cornea. The timing and indications for penetrating keratoplasty should be reconsidered by ophthalmologists; otherwise, the result may be evisceration or enucleation for endophthalmitis/panophthalmitis [14].

## Conclusion

*S. apiospermum* keratitis must be considered for both immunocompromised and immunocompetent patients, especially those with history of ocular trauma and contact lens use. Visual acuity can be increased by early diagnosis and aggressive treatment.

## Data Availability

Data sharing is not applicable to this article, as no datasets were generated or analyzed during the current study.

## Abbreviations

SDA: Sabouraud dextrose agar
BHI: Brain hard infusion
LPCB: Lactophenol cotton blue
MALDI TOF-MS: Matrix-assisted laser desorption ionization time-of-flight mass spectrometry
